# Body mass index and breast cancer survival: a Mendelian randomization analysis

**DOI:** 10.1093/ije/dyx131

**Published:** 2017-10-09

**Authors:** Qi Guo, Stephen Burgess, Constance Turman, Manjeet K Bolla, Qin Wang, Michael Lush, Jean Abraham, Kristiina Aittomäki, Irene L Andrulis, Carmel Apicella, Volker Arndt, Myrto Barrdahl, Javier Benitez, Christine D Berg, Carl Blomqvist, Stig E Bojesen, Bernardo Bonanni, Judith S Brand, Hermann Brenner, Annegien Broeks, Barbara Burwinkel, Carlos Caldas, Daniele Campa, Federico Canzian, Jenny Chang-Claude, Stephen J Chanock, Suet-Feung Chin, Fergus J Couch, Angela Cox, Simon S Cross, Cezary Cybulski, Kamila Czene, Hatef Darabi, Peter Devilee, W Ryan Diver, Alison M Dunning, Helena M Earl, Diana M Eccles, Arif B Ekici, Mikael Eriksson, D Gareth Evans, Peter A Fasching, Jonine Figueroa, Dieter Flesch-Janys, Henrik Flyger, Susan M Gapstur, Mia M Gaudet, Graham G Giles, Gord Glendon, Mervi Grip, Jacek Gronwald, Lothar Haeberle, Christopher A Haiman, Per Hall, Ute Hamann, Susan Hankinson, Jaana M Hartikainen, Alexander Hein, Louise Hiller, Frans B Hogervorst, Bernd Holleczek, Maartje J Hooning, Robert N Hoover, Keith Humphreys, David J Hunter, Anika Hüsing, Anna Jakubowska, Arja Jukkola-Vuorinen, Rudolf Kaaks, Maria Kabisch, Vesa Kataja, kConFab/AOCS Investigators, Julia A Knight, Linetta B Koppert, Veli-Matti Kosma, Vessela N Kristensen, Diether Lambrechts, Loic Le Marchand, Jingmei Li, Annika Lindblom, Sara Lindström, Jolanta Lissowska, Jan Lubinski, Mitchell J Machiela, Arto Mannermaa, Siranoush Manoukian, Sara Margolin, Federik Marme, John WM Martens, Catriona McLean, Primitiva Menéndez, Roger L Milne, Anna Marie Mulligan, Taru A Muranen, Heli Nevanlinna, Patrick Neven, Sune F Nielsen, Børge G Nordestgaard, Janet E Olson, Jose IA Perez, Paolo Peterlongo, Kelly-Anne Phillips, Christopher J Poole, Katri Pylkäs, Paolo Radice, Nazneen Rahman, Thomas Rüdiger, Anja Rudolph, Elinor J Sawyer, Fredrick Schumacher, Petra Seibold, Caroline Seynaeve, Mitul Shah, Ann Smeets, Melissa C Southey, Rob A E M Tollenaar, Ian Tomlinson, Helen Tsimiklis, Hans-Ulrich Ulmer, Celine Vachon, Ans MW van den Ouweland, Laura J Van’t Veer, Hans Wildiers, Walter Willett, Robert Winqvist, M Pilar Zamora, Georgia Chenevix-Trench, Thilo Dörk, Douglas F Easton, Montserrat García-Closas, Peter Kraft, John L Hopper, Wei Zheng, Marjanka K Schmidt, Paul DP Pharoah

**Affiliations:** 1 Cardiovascular Epidemiology Unit, Department of Public Health and Primary Care, University of Cambridge, Cambridge, UK,; 2 MRC Biostatistics Unit, University of Cambridge, Cambridge, UK,; 3 Program in Genetic Epidemiology and Statistical Genetics, Harvard T.H. Chan School of Public Health, Boston, MA, USA,; 4 Centre for Cancer Genetic Epidemiology, Department of Public Health and Primary Care, University of Cambridge, Cambridge, UK,; 5 Centre for Cancer Genetic Epidemiology, Department of Oncology, University of Cambridge, Cambridge, UK,; 6 Cambridge Experimental Cancer Medicine Centre, Cambridge, UK,; 7 Department of Clinical Genetics, Helsinki University Hospital, University of Helsinki, Helsinki, Finland,; 8 Fred A. Litwin Center for Cancer Genetics, Lunenfeld-Tanenbaum Research Institute of Mount Sinai Hospital, Toronto, Canada,; 9 Department of Molecular Genetics, University of Toronto, Toronto, Canada,; 10 Centre for Epidemiology and Biostatistics, Melbourne School of Population and Global Health, The University of Melbourne, Melbourne, Australia,; 11 Division of Clinical Epidemiology and Aging Research, German Cancer Research Center (DKFZ), Heidelberg, Germany,; 12 Division of Cancer Epidemiology, German Cancer Research Center (DKFZ), Heidelberg, Germany,; 13 Human Cancer Genetics Program, Spanish National Cancer Research Centre, Madrid, Spain,; 14 Centro de Investigación en Red de Enfermedades Raras (CIBERER), Valencia, Spain,; 15 Division of Cancer Epidemiology and Genetics, National Cancer Institute, Rockville, MD, USA,; 16 Department of Oncology, Helsinki University Hospital, University of Helsinki, Helsinki, Finland,; 17 Copenhagen General Population Study, Herlev and Gentofte Hospital, Copenhagen University Hospital, Herlev, Denmark,; 18 Department of Clinical Biochemistry, Herlev and Gentofte Hospital, Copenhagen University Hospital, Herlev, Denmark,; 19 Faculty of Health and Medical Sciences, University of Copenhagen, Copenhagen, Denmark,; 20 Division of Cancer Prevention and Genetics, Istituto Europeo di Oncologia, Milan, Italy,; 21 Department of Medical Epidemiology and Biostatistics, Karolinska Institutet, Stockholm, Sweden,; 22 Division of Preventive Oncology, German Cancer Research Center (DKFZ) and National Center for Tumor Diseases (NCT), Heidelberg, Germany,; 23 German Cancer Consortium (DKTK), German Cancer Research Center (DKFZ), Heidelberg, Germany,; 24 Division of Molecular Pathology, The Netherlands Cancer Institute – Antoni van Leeuwenhoek Hospital, Amsterdam, The Netherlands,; 25 Department of Obstetrics and Gynecology, University of Heidelberg, Heidelberg, Germany,; 26 Molecular Epidemiology Group, C080, German Cancer Research Center (DKFZ), Heidelberg, Germany,; 27 Cambridge Breast Unit and NIHR Cambridge Biomedical Research Centre, University of Cambridge NHS Foundation Hospitals, Cambridge, UK,; 28 Breast Cancer Functional Genomics Laboratory, Cancer Research UK Cambridge Institute, University of Cambridge, Li Ka Shing Centre, UK,; 29 Department of Biology, University of Pisa, Pisa, Italy,; 30 Genomic Epidemiology Group, German Cancer Research Center (DKFZ), Heidelberg, Germany,; 31 University Cancer Center Hamburg (UCCH), University Medical Center Hamburg-Eppendorf, Hamburg, Germany,; 32 Department of Laboratory Medicine and Pathology, Mayo Clinic, Rochester, MN, USA,; 33 Academic Unit of Molecular Oncology, Department of Oncology and Metabolism, University of Sheffield, Sheffield, UK,; 34 Academic Unit of Pathology, Department of Neuroscience, University of Sheffield, Sheffield, UK,; 35 Department of Genetics and Pathology, Pomeranian Medical University, Szczecin, Poland,; 36 Department of Pathology, Leiden University Medical Center, Leiden, The Netherlands,; 37 Department of Human Genetics, Leiden University Medical Center, Leiden, The Netherlands,; 38 Epidemiology Research Program, American Cancer Society, Atlanta, GA, USA,; 39 Cancer Sciences Academic Unit, Faculty of Medicine, University of Southampton, Southampton, UK,; 40 Institute of Human Genetics, University Hospital Erlangen, Friedrich-Alexander University Erlangen-Nuremberg, Comprehensive Cancer Center Erlangen-EMN, Erlangen, Germany,; 41 Genomic Medicine, Manchester Academic Health Science Centre, University of Manchester, Central Manchester Foundation Trust, St. Mary's Hospital, Manchester, UK,; 42 Department of Gynaecology and Obstetrics, University Hospital Erlangen, Friedrich-Alexander University Erlangen-Nuremberg, Comprehensive Cancer Center Erlangen-EMN, Erlangen, Germany,; 43 David Geffen School of Medicine, Department of Medicine Division of Hematology and Oncology, University of California at Los Angeles, Los Angeles, CA, USA,; 44 Usher Institute of Population Health Sciences and Informatics, The University of Edinburgh Medical School, Edinburgh, UK,; 45 Institute for Medical Biometrics and Epidemiology, University Medical Center Hamburg-Eppendorf, Hamburg, Germany,; 46 Department of Cancer Epidemiology, Clinical Cancer Registry, University Medical Center Hamburg-Eppendorf, Hamburg, Germany,; 47 Department of Breast Surgery, Herlev and Gentofte Hospital, Copenhagen University Hospital, Herlev, Denmark,; 48 Cancer Epidemiology & Intelligence Division, Cancer Council Victoria, Melbourne, Australia,; 49 Department of Surgery, Oulu University Hospital, University of Oulu, Oulu, Finland,; 50 Department of Preventive Medicine, Keck School of Medicine, University of Southern California, Los Angeles, CA, USA,; 51 Molecular Genetics of Breast Cancer, German Cancer Research Center (DKFZ), Heidelberg, Germany,; 52 Department of Biostatistics & Epidemiology, University of Massachusetts, Amherst, Amherst, MA, USA,; 53 Translational Cancer Research Area, University of Eastern Finland, Kuopio, Finland,; 54 Institute of Clinical Medicine, Pathology and Forensic Medicine, University of Eastern Finland, Kuopio, Finland,; 55 Imaging Center, Department of Clinical Pathology, Kuopio University Hospital, Kuopio, Finland,; 56 Warwick Clinical Trials Unit, University of Warwick, Coventry, UK,; 57 Family Cancer Clinic, The Netherlands Cancer Institute – Antoni van Leeuwenhoek Hospital, Amsterdam, The Netherlands,; 58 Saarland Cancer Registry, Saarbrücken, Germany,; 59 Department of Medical Oncology, Family Cancer Clinic, Erasmus MC Cancer Institute, Rotterdam, The Netherlands,; 60 Department of Epidemiology, Harvard T.H. Chan School of Public Health, Boston, MA, USA,; 61 Department of Oncology, Oulu University Hospital, University of Oulu, Oulu, Finland,; 62 Central Finland Health Care District, Jyväskylä Central Hospital, Jyväskylä, Finland,; 63 Division of Cancer Medicine, Peter MacCallum Cancer Center, Melbourne, Australia,; 64 Prosserman Centre for Health Research, Lunenfeld-Tanenbaum Research Institute of Mount Sinai Hospital, Toronto, Canada,; 65 Division of Epidemiology, Dalla Lana School of Public Health, University of Toronto, Toronto, Canada,; 66 Department of Surgical Oncology, Family Cancer Clinic, Erasmus MC Cancer Institute, Rotterdam, The Netherlands,; 67 Department of Cancer Genetics, Institute for Cancer Research, Oslo University Hospital Radiumhospitalet, Oslo, Norway,; 68 Institute of Clinical Medicine, Faculty of Medicine, University of Oslo, Oslo, Norway,; 69 Department of Clinical Molecular Biology, Oslo University Hospital, University of Oslo, Oslo, Norway,; 70 VIB Center for Cancer Biology, VIB, Leuven, Belgium,; 71 Laboratory for Translational Genetics, Department of Human Genetics, University of Leuven, Leuven, Belgium,; 72 University of Hawaii Cancer Center, Honolulu, HI, USA,; 73 Department of Molecular Medicine and Surgery, Karolinska Institutet, Stockholm, Sweden,; 74 Department of Epidemiology, University of Washington School of Public Health, Seattle, WA, USA,; 75 Department of Cancer Epidemiology and Prevention, M. Sklodowska-Curie Memorial Cancer Center – Oncology Institute, Warsaw, Poland,; 76 Unit of Medical Genetics, Department of Medical Oncology and Hematology, Fondazione IRCCS (Istituto di Ricovero e Cura a Carattere Scientifico) Istituto Nazionale dei Tumori (INT), Milan, Italy,; 77 Department of Oncology – Pathology, Karolinska Institutet, Stockholm, Sweden,; 78 National Center for Tumor Diseases, University of Heidelberg, Heidelberg, Germany,; 79 Anatomical Pathology, The Alfred Hospital, Melbourne, Australia,; 80 Servicio de Anatomía Patológica, Hospital Monte Naranco, Oviedo, Spain,; 81 Department of Laboratory Medicine and Pathobiology, University of Toronto, Toronto, Canada,; 82 Laboratory Medicine Program, University Health Network, Toronto, Canada,; 83 Department of Obstetrics and Gynecology, Helsinki University Hospital, University of Helsinki, Helsinki, Finland,; 84 Leuven Multidisciplinary Breast Center, Department of Oncology, Leuven Cancer Institute, University Hospitals Leuven, Leuven, Belgium,; 85 Department of Health Sciences Research, Mayo Clinic, Rochester, MN, USA,; 86 Servicio de Cirugía General y Especialidades, Hospital Monte Naranco, Oviedo, Spain,; 87 Sir Peter MacCallum Department of Oncology, The University of Melbourne, Melbourne, Australia,; 88 Department of Medicine, St Vincent's Hospital, The University of Melbourne, Fitzroy, Australia,; 89 Laboratory of Cancer Genetics and Tumor Biology, Cancer and Translational Medicine Research Unit, Biocenter Oulu, University of Oulu, Oulu, Finland,; 90 Laboratory of Cancer Genetics and Tumor Biology, Northern Finland Laboratory Centre Oulu, Oulu, Finland,; 91 Unit of Molecular Bases of Genetic Risk and Genetic Testing, Department of Research, Fondazione IRCCS (Istituto Di Ricovero e Cura a Carattere Scientifico) Istituto Nazionale dei Tumori (INT), Milan, Italy,; 92 Division of Genetics and Epidemiology, The Institute of Cancer Research, London, UK,; 93 Institute of Pathology, Staedtisches Klinikum Karlsruhe, Karlsruhe, Germany,; 94 Research Oncology, Guy's Hospital, King's College London, London, UK,; 95 Department of Epidemiology and Biostatistics, Case Western Reserve University, Cleveland, OH, USA,; 96 Department of Pathology, The University of Melbourne, Melbourne, Australia,; 97 Department of Surgery, Leiden University Medical Center, Leiden, The Netherlands,; 98 Wellcome Trust Centre for Human Genetics and Oxford NIHR Biomedical Research Centre, University of Oxford, Oxford, UK,; 99 Frauenklinik der Stadtklinik Baden-Baden, Baden-Baden, Germany,; 100 Department of Clinical Genetics, Erasmus University Medical Center, Rotterdam, The Netherlands,; 101 Department of Nutrition, Harvard T.H. Chan School of Public Health, Boston, MA, USA,; 102 Servicio de Oncología Médica, Hospital Universitario La Paz, Madrid, Spain,; 103 Department of Genetics and Computational Biology, QIMR Berghofer Medical Research Institute, Brisbane, Australia,; 104 Gynaecology Research Unit, Hannover Medical School, Hannover, Germany,; 105 Division of Epidemiology, Department of Medicine, Vanderbilt Epidemiology Center, Vanderbilt-Ingram Cancer Center, Vanderbilt University School of Medicine, Nashville, TN, USA and; 106 Division of Psychosocial Research and Epidemiology, The Netherlands Cancer Institute – Antoni van Leeuwenhoek Hospital, Amsterdam, The Netherlands,; 107 IFOM, The FIRC (Italian Foundation for Cancer Research) Institute of Molecular Oncology, Milan, Italy

**Keywords:** Body mass index, breast cancer survival, Mendelian randomization, epidemiology, genetics

## Abstract

**Background:**

There is increasing evidence that elevated body mass index (BMI) is associated with reduced survival for women with breast cancer. However, the underlying reasons remain unclear. We conducted a Mendelian randomization analysis to investigate a possible causal role of BMI in survival from breast cancer.

**Methods:**

We used individual-level data from six large breast cancer case-cohorts including a total of 36 210 individuals (2475 events) of European ancestry. We created a BMI genetic risk score (GRS) based on genotypes at 94 known BMI-associated genetic variants. Association between the BMI genetic score and breast cancer survival was analysed by Cox regression for each study separately. Study-specific hazard ratios were pooled using fixed-effect meta-analysis.

**Results:**

BMI genetic score was found to be associated with reduced breast cancer-specific survival for estrogen receptor (ER)-positive cases [hazard ratio (HR) = 1.11, per one-unit increment of GRS, 95% confidence interval (CI) 1.01–1.22, *P* = 0.03). We observed no association for ER-negative cases (HR = 1.00, per one-unit increment of GRS, 95% CI 0.89–1.13, *P* = 0.95).

**Conclusions:**

Our findings suggest a causal effect of increased BMI on reduced breast cancer survival for ER-positive breast cancer. There is no evidence of a causal effect of higher BMI on survival for ER-negative breast cancer cases.

**Key Messages**

- Observational studies have reported an association between elevated body mass index (BMI) and reduced survival for women with breast cancer. However, the causal nature of the association is unclear.
- We conducted a large Mendelian randomization analysis in order to examine a potential causal effect of BMI on breast cancer survival, using both individual genotype data and summary data.
- Our study provides evidence that the reported association between BMI and survival for estrogen receptor-positive breast cancer is likely to be causal.

## Introduction

Breast cancer is the most common form of cancer for women worldwide.^1^ There is substantial variation in survival outcomes between patients. Some of this variation can be explained by established clinico-pathological factors including clinical stage, tumour grade and the molecular phenotype of the tumour. However, other factors such as germline genetic variation^2^ and lifestyle factors may also be important. The association between body mass index (BMI) and survival has been investigated in many studies with increased BMI being associated with a reduced survival,^3–11^ with some studies reporting an association limited to estrogen receptor (ER)-positive disease.^12–15^ Whether this association is causal or simply due to confounding by other factors remains unclear.

Mendelian randomization (MR)^16^^,^^17^ has become an established method used to estimate the causal relationship between an exposure and an associated outcome using data on inherited genetic variants that influence exposure status. Genetic variants are attractive as candidate instrumental variables because they are randomly assigned at conception and are not affected by potential environmental confounding factors. The use of germline genetic variants as instruments for modifiable exposures has the potential to avoid some of the limitations of conventional observational epidemiology for making causal inferences.^18^ Recent genome-wide association studies have identified multiple loci associated with BMI,^19^ enabling investigation of a possible causal role of BMI in breast cancer outcomes using an MR approach.

The aim of this study was to utilize germline genotype data for genetic variants known to be associated with BMI, in a breast cancer case-cohort to evaluate the association between BMI and breast cancer survival in an unbiased way. There are three assumptions under which genetic variants provide valid instrumental variables for the effect of BMI on breast cancer survival: first, the genetic variants are associated with BMI; second, the variants are not associated with any confounder of the BMI-breast cancer survival association (pleiotropy); third, the variants are conditionally independent of the survival, given the BMI and confounders (exclusion restriction).

## Methods

We included six datasets where a genotyping array providing genome-wide coverage of common genetic variation had been used to genotype multiple breast cancer case-cohorts in populations of European ancestry (COGS, CGEMS, METABRIC, PG-SNPs, SASBAC and UK2). A summary of these case-cohorts has been described in detail previously.^2^ The characteristics of the studies used in our analysis are summarized in Table S1 (available as Supplementary data at *IJE* online). Genotypes for common variants across the genome were imputed using a reference panel from the 1000 Genomes Project (March 2012) for each dataset. All patients provided written informed consent, and each study was approved by the relevant institutional review board. Data on age at diagnosis, vital status, breast cancer-specific mortality, follow-up time, time between diagnosis and blood draw, lymph node status, histological grade, tumour size and estrogen receptor status were also available. In addition, some case-cohorts from the COGS study provided data on height and weight (self-reported) at date closest to diagnosis (cases) or study entry (controls) for 65 582 participants. BMI was calculated as weight in kilograms divided by height in metres squared (kg/m^2^).

### Calculation of BMI genetic risk score

The Genetic Investigation of Anthropometric Traits (GIANT) consortium involving over 300 000 individuals of European descent has reported 97 common variants associated with BMI, of which three were only associated with BMI for men.^19^ We used the genotype data described above to construct the BMI genetic risk score (GRS) based on 94 BMI-associated genetic variants . The BMI genetic risk score is given by the sum of the weighted imputed allele doses (number of risk alleles carried) where the weights are the reported beta-coefficients for association with BMI. The manuscript^19^ presented the results as the number of standard deviations increase in BMI per allele. We therefore transformed these to the increase in BMI per allele. The imputation r^2^ of all 94 single nucleotide polymorphisms (SNPs) in the breast cancer dataset is greater than 0.4.

### Statistical analysis

We verified the first assumption of Mendelian randomization by evaluating the association between BMI GRS and BMI in a set of control subjects from the COGS study. MR analysis was performed using Cox proportional hazard models, to evaluate the associations of the BMI genetic risk scores with breast cancer-specific mortality based on 36 210 cases with 2475 events over 170 504 person-years of follow-up. The date of diagnosis was used to calculate time-to-event with follow-up being censored at death, last follow-up or 10 years, whichever came sooner. As several studies include prevalent cases, the date of study entry was used to determine time under observation in order to adjust for the potential bias of prevalent cases in a prospectively recruiting study (left-truncation).^20^ All analyses were performed for each study separately, and summary statistics were obtained using a fixed-effect meta-analysis. We also conducted MR subtype-specific analysis for 5683 ER-negative cases (679 events) and 22 567 ER-positive cases (1161 events) (Table S1).

We assessed the relationship between BMI GRS and breast cancer survival using summary statistics for the association of each BMI- associated SNP with survival, for each dataset. We used both an inverse-variance weighted method and a likelihood-based method^21^ to estimate the association. Several clinico-pathological factors are known to be associated with survival. Rather than being true potential confounders of any relationship between BMI and survival, these factors should be considered as intermediates. Nevertheless, in order to evaluate the second assumption of MR, we tested for association between BMI-associated SNPs and node status, tumour size and histological grade. Alternatively, it is possible that smoking behaviour might mediate the true casual mechanisms for the association between BMI and breast cancer survival. We examined therefore the potential associations between smoking behaviour (measured as self-reported total pack-years smoked) and survival and between GRS and smoking behaviour. Pleiotropic effects of the BMI SNPs on unmeasured confounders may also violate the assumption. The role of directional pleiotropy was assessed using Egger regression on the summary statistics of association for each BMI-associated SNP with survival.^22^ Egger regression is a modified form of standard inverse-variance weighted meta-analysis. When applied to MR analyses, the slope of the Egger regression provides an estimate of the causal effect, and the estimated value of the intercept can be interpreted as an estimate of the average pleiotropic effect across all the genetic variants.^23^ All analyses were performed using R (R project for Statistical Computing).

### Results

We observed strong positive associations between the BMI GRS and observed BMI using a set of 28 190 controls from the COGS study. A one-unit increase in GRS corresponds to a 0.94 kg/m^2^ (95% CI 0.85–1.03, *P* = 4.16 × 10^−99^) increase in BMI and explained 1.6% of the BMI variance (F statistic = 450). Self-reported BMI was significantly associated with breast cancer survival for both ER-negative and ER-positive disease in the COGS data (Table 1). Both associations were attenuated after adjustment for tumour grade, nodal status and tumour size.

**Table 1:** Association between BMI genetic risk score and survival for ER-positive and ER-negative breast cancer

	ER-negative		ER-positive	
	HR (95% CI)	*P*	HR (95%CI)	*P*
Observational estimate^a^				
Unadjusted	1.02 (1.01–1.04)	0.01	1.03 (1.02–1.04)	2.37 × 10^−5^
Adjusted for nodes, size and grade	1.00 (0.97–1.02)	0.77	1.02 (1.00–1.05)	0.05
Individual-level data MR analysis				
GRS	1.00 (0.89–1.13)	0.95	1.11 (1.01–1.22)	0.03
Summary results MR analysis				
GRS IVW^b^	1.01 (0.91–1.12)	0.91	1.11 (1.01–1.21)	0.02
GRS likelihood-based	1.01 (0.91–1.12)	0.91	1.11 (1.02–1.21)	0.02
GRS Egger regression	0.91 (0.70–1.18)	0.46	1.11 (0.89–1.38)	0.36

^a^Association between self-reported BMI and survival (HR per unit increase in BMI).

^b^Inverse-variance weighted.

We performed MR analysis for all available ER-negative and ER-positive breast cancer cases. The GRS was found to be associated with reduced breast cancer-specific survival for ER-positive cases with hazard ratio (HR) of 1.11 (95% CI = 1.01–1.22, *P* = 0.03) per one-unit increment of the GRS (Table 1). In order to evaluate whether this association varied by menopausal status, we compared the estimates for GRS for premenopausal (defined as age at diagnosis < 50 years) and postmenopausal (age at diagnosis ≥ 50 years) women with ER-positive breast cancer, using data from the COGS study. We found no evidence for a difference in the hazard ratios (*P* = 0.93).

No significant association with genetic score was observed for ER-negative cases (HR = 1.00, 95% CI 0.89–1.13; Table 1). This indicates that the observed association between BMI and breast cancer survival for ER-negative cases might not be causal. However, we had only 38% power to detect the same magnitude of association as that observed for ER-positive disease with a type I error of 5%.^24^ The number of events would need to be approximately 2000 for a power of 80% in ER-negative cases (Supplementary Figure 1, available as Supplementary data at *IJE* online). The differences between the estimated associations with genetic score for ER-positive and ER-negative were not significant (*P* = 0.07). The association between BMI and breast cancer survival was also evaluated using standard inverse-variance weighted meta-analysis of summary statistics for the association of each BMI-associated SNP with survival. The results were similar to those based on individual-level data (Table 1).

**Figure 1:**
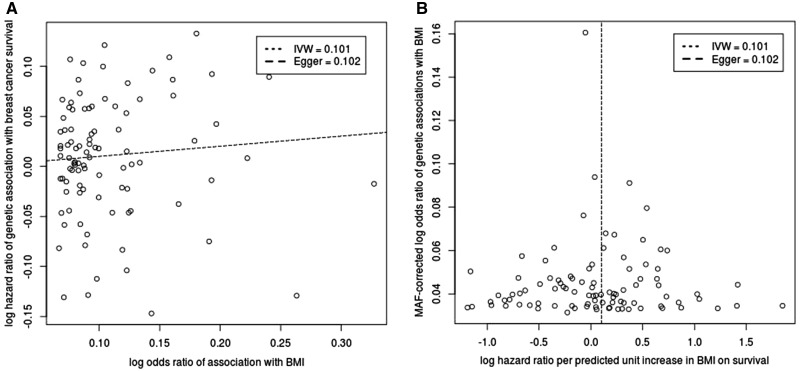
Genetic associations of BMI GRS and breast cancer survival for ER-positive cases. (A) Scatter plot of log hazard ratio of genetic associations with breast cancer survival for ER-positive cases against log odds ratio of association with BMI. Slope of the regression lines: inverse variance-weighted (dotted) and MR-Egger (dashed) provide an estimate of the predicted log hazard ratio per unit increase in BMI. (B) Funnel plot of minor allele frequency (MAF)-corrected log odds ratio of genetic associations with BMI against log hazard ratio per predicted unit increase in BMI on breast cancer survival for each genetic variant individually.

In order to test the validity of the exclusion restriction assumption, we compared the results of a standard inverse-variance weighted regression with the Egger regression for the SNPs in the GRS (Figure 1A). The slope of the inverse-variance weighted regression was 0.10 (95% CI 0.02–0.19) which was similar to that from the Egger regression 0.10 (95% CI 0.11–0.32). The intercept from the Egger regression was not significantly different from zero (−0.0002, *P*-value = 0.99), suggesting no overall directional pleiotropy. A funnel plot of the minor allele frequency-corrected genetic associations with the BMI against the individual causal effect estimates for each SNP shows little evidence for asymmetry (Figure 1B).

We tested each GRS SNP for association with either node status or grade or tumour size or stage. Sixteen of the BMI SNPs were associated with one or more of these variables. We then repeated the individual data MR analysis using a GRS-78 that excluded these SNPs. The magnitudes of the associations with ER-positive breast cancer were similar to those for the results based on all the BMI SNPs (GRS-78: HR = 1.10, 95% CI 1.00–1.22, *P* = 0.06).

We explored a potential complex relationship between smoking behaviour, BMI and prognosis by investigating the association between BMI GRS and smoking behaviour and between smoking behaviour and prognosis. There was a very weak correlation between GRS and number of pack-years smoked (correlation coefficient = 0.017, *P* = 0.004). However, there was no association between smoking and prognosis (*P* = 0.47 and 0.79 for ER-positive and ER-negative disease, respectively). It is unlikely that the association between smoking behaviour and BMI can explain the association between BMI GRS and prognosis.

## Discussion

We conducted a large Mendelian randomization analysis in order to examine a potential causal effect of BMI on breast cancer survival, using both individual data and summary data. We constructed a weighted BMI genetic score comprising 94 BMI-associated genetic variants identified in genome-wide association studies as instrumental variables. We also used an inverse-variance weighted method and likelihood-based method to evaluate the combined association of BMI-associated SNPs with breast cancer survival. The results from the summarized data were in agreement with the results from two-stage regression based on individual-level genotype data. Our findings suggest a possible causal association between increased BMI and reduced breast cancer survival for ER-positive cases. This provides consistent evidence, along with other findings, that increased BMI has been repeatedly associated with ER-positive breast cancer.

A limitation of the analysis is that, even if the genetic variants are not associated with confounders of the relationship between BMI and breast cancer survival for the population as a whole (that is, the genetic variants are valid instrumental variables for the population), the genetic variants may be associated with these confounders for the subpopulation of breast cancer patients. This is due to conditioning on a collider: if BMI is a causal risk factor for breast cancer risk, then conditioning on breast cancer risk (by only including breast cancer patients in the analysis) means that all common causes of breast cancer risk (including the genetic variants and confounders) are conditionally associated. In simple terms, even if genetic variants are distributed randomly in the population as a whole, they are not necessary randomly distributed in the ascertained population of breast cancer patients. This may lead to bias in the analysis, although it is unclear how serious this bias might be. In order to evaluate the potential for collider bias, we performed a simulation study in which we simulated data on a genetic risk score and an exposure (BMI in our example) for 100 000 individuals. For each individual, we simulated whether that individual had a positive breast cancer diagnosis as a binomial random variable. For each individual with a positive breast cancer diagnosis, we simulated the time-to-event for breast cancer progression as an exponential random variable. The genetic risk score was simulated as a normally distributed random variable, as was the confounder (assumed unmeasured), and the independent error term. The probability of breast cancer diagnosis was modelled as a function of the exposure. This leads to the collider (selection) bias: individuals with a breast cancer diagnosis (and therefore eligible for the Mendelian randomization analysis) will have higher average levels of the exposure and confounder than those not included. While collider bias was observed for extreme values of the effect of the risk factor on disease status, it was not observed for values that are in line with the effect of BMI on breast cancer diagnosis as observed in previous investigations. Hence, while we would be cautious not to generalize the result of this limited simulation study to other analysis contexts, in this case there seemed to be little potential for bias and type 1 error rate inflation to arise due to collider bias.

While our results suggest a causal association between BMI and survival for women with ER-positive breast cancer, BMI is, in itself, a complex phenotype. It is conceivable that more specific phenotypes related to body fat composition and distribution might be better predictors of outcome. Untangling such complex relationships with survival will require data on the association between germline genetic variation and specific body fat composition and distribution phenotypes. Potential mechanisms underlying effects of obesity on breast cancer survival are mediators such as members of the insulin/insulin-like growth factor family, adipocytokines secreted from adipose tissue and inflammatory cytokines.^23^

Our study, based on data from multiple large-scale genetic association studies of breast cancer, provides evidence that the reported association between BMI and survival for ER-positive breast cancer is likely to be causal. This suggests that BMI reduction in overweight women with ER-positive breast cancer might improve clinical outcomes.

## Supplementary Data

Supplementary data are available at *IJE* online.

## Supplementary Material

Supplementary Data
