# Supplementary material for: Body mass index and breast cancer survival: a Mendelian randomization analysis
Source: Int J Epidemiol. 2017 Oct 9;46(6):1814–22. doi: 10.1093/ije/dyx131 (PMC5837506; doi:10.1093/ije/dyx131)
Supplement: Supplementary Data [file dyx131_supplementary_data.pdf]

Supplementary Data

Guo et al. Body Mass Index and Breast Cancer Survival: A Mendelian Randomisation Analysis

List of Table

Table S1 Summary of samples included in the datasets contributing data for analysis

List of Figure

Supplementary Figure 1: Power plot of MR analysis for ER-negative breast cancer cases.

Funding

Acknowledgements

Table S1 Summary of samples included in the datasets contributing data for analysis

Study	All cases	ER-positive	ER-negative
	N (breast cancer deaths)	N (breast cancer deaths)	N (breast cancer deaths)
COGS	29,360 (1790)	20,605 (942)	4,926 (558)
CGEMS	1,145 (93)	--	--
SASBAC	787 (69)	483 (44)	108 (9)
UK2	2,763 (233)	--	
Metabric	369 (86)	291 (59)	63 (25)
PG-SNPs	1,786 (204)	1,188 (116)	586 (87)
**Total**	**36,210 (2,475)**	**22,567 (1,161)**	**5,683 (679)**

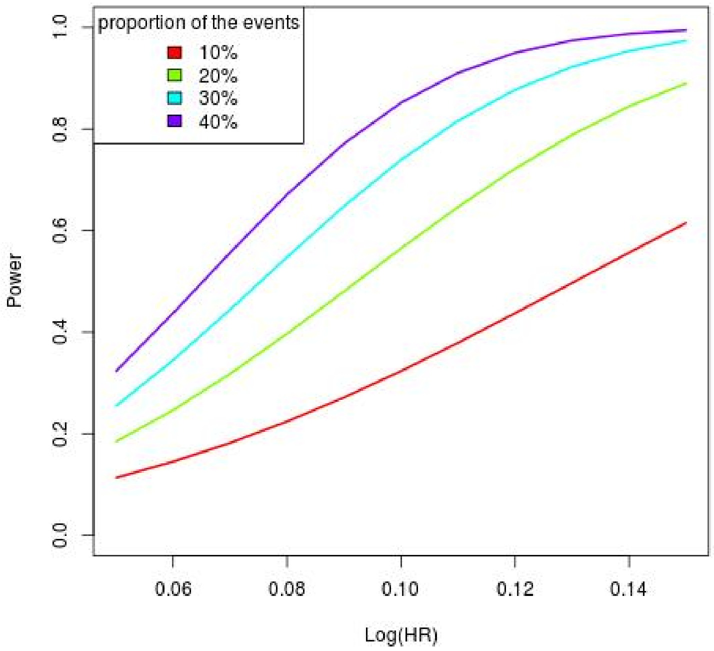

Supplementary Figure 1: Power plot of MR analysis for ER-negative breast cancer cases.

Funding

Higher level funding

The COGS project was funded through a European Commission's Seventh Framework Programme grant (agreement number 223175 - HEALTH-F2-2009-223175). The Breast Cancer Association Consortium (BCAC) is funded by Cancer Research-UK (C1287/A10118 and C1287/A12014). Meetings of the BCAC have been funded by the European Union COST programme (BM0606). ELAN Program of the University Hospital Erlangen (BBCC). Funds from Italian citizens who allocated the 5/1000 share of their tax payment in support of the Fondazione IRCCS Istituto Nazionale Tumori, according to Italian laws (INT-Institutional strategic projects “5x1000”).

Personal support

D.F.E is a Principal Research Fellow of Cancer Research UK. J.L.H is a National Health and Medical Research Council (NHMRC) Australia Fellow. S.B is supported by a fellowship from the Wellcome Trust (100114). M.C.S. is a NHMRC Senior Research Fellow. G.C.T is an NHMRC Senior Principal Research Fellow. D.L is supported by the FWO and the KULPFV/10/016-SymBioSysII. J.L is a UNESCO-L'Oréal International Fellow. I.L.A. holds the Anne and Max Tanenbaum Chair in Molecular Medicine at Mount Sinai Hospital and the University of Toronto. K.A.P. is an Australian National Breast Cancer Foundation Practitioner Fellow.

Funding of constituent studies

These are listed by funding agency, with each grant number in parentheses

Funding of the constituent studies was provided by:

Academy of Finland (266528);

Addenbrookes Charitable Trust;

Agency for Science, Technology and Research of Singapore;

Asociación Española Contra el Cáncer and the Fondo de Investigación Sanitario (PI11/00923 , PI08/1120);

Baden Württemberg Ministry of Science, Research and Arts;

Breast Cancer Campaign (2009PR42);

Breast Cancer Research Foundation;

Canadian Institutes of Health Research (CIHR Team in Familial Risks of Breast Cancer program);

Cancer Australia and National Breast Cancer Foundation Priority-driven Collaborative Cancer Research Scheme (#809195);

Cancer Councils of New South Wales, Victoria, Tasmania and South Australia;

Cancer Foundation of Western Australia;

Cancer Fund of North Savo;

Cancer Research UK (A7572, A10124, A11699, A16561);

Chief Physician Johan Boserup and Lise Boserup Fund;

Danish Medical Research Council;

Deutsche Krebshilfe (70-2892-BR I, PBZ_KBN_122/P05/2004);

Dietmar-Hopp Foundation;

Dutch Cancer Society (1997-1505, 2004-3124, 2007-3839, 2009-4318, 2009-4363);

Dutch government (NWO 184.021.007);

Dutch National Genomics Initiative;

ELAN-Fond of the University Hospital of Erlangen;

Federal Ministry of Education and Research, Germany (01KH0402);

Finnish Cancer Society;

Fondazione IRCCS Istituto Nazionale Tumori;

Genome Spain Foundation;

German Cancer Aid (Deutsche Krebshilfe);

German Cancer Research Center (DKFZ);

Hamburg Cancer Society;

Helmholtz Society;

Helsinki University Central Hospital Research Fund,

Italian Association for Cancer Research(AIRC);

Kuopio University Hospital special Government Funding;

National Health and Medical Research Council of Australia (209057, 251553, 504711, 454508, 288704, 145684);

NIHR Biomedical Research Centre Biomedical Research Centre;

Nordic Cancer Union;

Märit and Hans Rausings Initiative Against Breast Cancer; Nordic Cancer Union;

Polish Foundation of Science (PBZ_KBN_122/P05/2004);

Queensland Cancer Fund;

Red Temática de Investigación Cooperativa en Cáncer;

Sheffield Experimental Cancer Medicine Centre

Sigrid Juselius Foundation;

Susan G. Komen Breast Cancer Foundation;

Stichting tegen Kanker (232-2008 and 196-2010);

United States National Institutes of Health (BBMRI-NL-CP16, CA69638, CA69417, CA06503, CA116201, CA122340, CA128978, CA63464, CA54281, CA098758, CA132839, CA164920, CA164973, CA98216 , CA098233, CA148065, CA98710, CA98758, and Intramural Research Program of National Institutes of Health and National Cancer Institute);

UK National Institute for Health Research Biomedical Research Centres at the University of Cambridge;

NIHR Biomedical Research Centre; Guy's & St. Thomas' NHS Foundation Trust in partnership with King's College London, and University of Oxford;

University of Eastern Finland strategic funding;

Victorian Health Promotion Foundation

Victorian Breast Cancer Research Consortium;

Yorkshire Cancer Research (S295, S299, S305PA)

Acknowledgements

The study sponsors had no role in the design of the study; the collection, analysis, and interpretation of the data; the writing of the manuscript; and the decision to submit the manuscript for publication.

This study would not have been possible without the contributions of the following: D. C. Tessier, F. Bacot, D. Vincent, S. LaBoissière and F. Robidoux and the staff of the genotyping unit, (Genome Quebec); J. Stone, S. McBean, J. Hadlington, A. Mustafa and K. Cook (Illumina); M. Angelakos, J. Maskiell, G. Dite (ABCFS); S. Cornelissen, R. van Hien, L. Braaf, F. Hogervorst, S. Verhoef, E. Rutgers, F. Atsma (ABCS); N. McInerney, G. Colleran, A. Rowan, A. Jones (BIGGS); B Henderson (BPC3); P. Bugert (BSUCH); D. U. Andersen, M. B. Arnadottir, A. Bank, D. K. Hansen (CGPS); G. Pita, C. Alonso, D. Herrero, N. Álvarez, P. Zamora, P. Menendez (CNIO-BCS); C. Stegmaier, H. Ziegler, S. Wolf, B. Holleczek, V. Hermann, K.-U. Saum (ESTHER); D. Greco, K. von Smitten, I. Erkkilä (HEBCS); E. Myöhänen, H. Kemiläinen (KBCP); G. Peuteman, D. Smeets, T. Van Brussel, K. Corthouts (LMBC); Judith Heinz, Nadia Obi, Alina Vrieling, Sabine Behrens, Ursula Eilber, Muhabbet Celik, Til Olchers (MARIE); B. Peissel, J. Azzollini, D. Zaffaroni, M. Barile, I. Feroce, V. Pensotti (MBCSG); K. Mononen, M. Otsukka (OBCS); T. Selander, N. Weerasooriya, G. Glendon (OFBCR); E. Krol-Warmerdam, J. Blom. J. Molenaar (ORIGO); L. Brinton, M. Sherman, N. Szeszenia-Dabrowska, B. Peplonska, W. Zatonski, P. Chao, M. Stagner (PBCS); P. Bos, J. Blom, E. Crepin, E. Huijskens, A. Heemskerk (RBCS); S. Higham, Ian Brock, Sabapathy Balasubramanian, Malcolm W.R.Reed (SBCS); J.Young, C. Twelves, AL. Vallier, S. Ingle, R. Hardy (PGSNPS); Matthias Rübner, Silke Landrith, Sonja Oeser (BBCC).
